# Serotonin enhances the impact of health information on food choice

**DOI:** 10.3758/s13415-016-0496-2

**Published:** 2017-01-23

**Authors:** Ivo Vlaev, Molly J. Crockett, Luke Clark, Ulrich Müller, Trevor W. Robbins

**Affiliations:** 1 Warwick Business School, University of Warwick, Coventry, UK; 2 Department of Experimental Psychology, University of Oxford, Oxford, UK; 3 Centre for Gambling Research at UBC, Department of Psychology, University of British Columbia, Vancouver, BC Canada; 4 Department of Psychiatry, University of Cambridge, Cambridge, UK; 5 Adult ADHD Service, Cambridgeshire & Peterborough NHS Foundation Trust (CPFT), Cambridge, UK; 6 Department of Psychology, and Behavioural and Clinical Neuroscience Institute, University of Cambridge, Cambridge, UK

**Keywords:** Serotonin, Motivation, Action control, Decision making, Food choice

## Abstract

Serotonin has been implicated in promoting self-control, regulation of hunger and physiological homeostasis, and regulation of caloric intake. However, it remains unclear whether the effects of serotonin on caloric intake reflect purely homeostatic mechanisms, or whether serotonin also modulates cognitive processes involved in dietary decision making. We investigated the effects of an acute dose of the serotonin reuptake inhibitor citalopram on choices between food items that differed along taste and health attributes, compared with placebo and the noradrenaline reuptake inhibitor atomoxetine. Twenty-seven participants attended three sessions and received single doses of atomoxetine, citalopram, and placebo in a double-blind randomised cross-over design. Relative to placebo, citalopram increased choices of more healthy foods over less healthy foods. Citalopram also increased the emphasis on health considerations in decisions. Atomoxetine did not affect decision making relative to placebo. The results support the hypothesis that serotonin may influence food choice by enhancing a focus on long-term goals. The findings are relevant for understanding decisions about food consumption and also for treating health conditions such as eating disorders and obesity.

People often must decide between an option with higher overall long-term value and a tempting but ultimately less beneficial option. Optimal decision making in such situations, including food choice, requires value computation and action control (Hare, Camerer, & Rangel, 2009). A growing body of literature suggests that trade-offs between short-term and long-term goals are mediated by interactions between the prefrontal cortex and subcortical structures (van den Bos et al., 2013; Dalley, Everitt, & Robbins, 2011), and that the monoamine neurotransmitters serotonin (5-HT) and noradrenaline (NA) may modulate the activity in these systems (Robbins, 2007).

In dietary decision making, there is abundant evidence implicating serotonin in regulation of appetite and caloric intake (see Carek & Dickerson, 2012; Halford & Harrold, 2011; Jason et al., 2011). However, it is unclear whether these effects on caloric intake reflect purely homeostatic mechanisms, or whether there are also cognitive mechanisms at play. If there are cognitive mechanisms involved, then this would have potential implications for designing treatments for obesity; for example, serotonin drugs may be more effective if combined with cognitive interventions. Furthermore, Doucerain and Fellows (2012) review the literature on dietary decision making and argue that previous research has not used reliable measures of food intake and choice, which prevents clear understanding of the underlying cognitive mechanisms. A similar argument can be made about previous research on the effect of serotonin on food intake, which has focused mostly on studying the quantity of calories consumed rather than on the psychological and neural mechanisms involved in dietary decisions. Such deeper understanding would require novel and theoretically informed experimental designs.

There is evidence that serotonin affects cognitive processes crucial for action control (Chamberlain et al., 2006; Hyman, 2011) and value-based decision making (Boureau & Dayan, 2011; Seymour et al., 2012). However, the precise role of serotonin in action control is not well understood (Clark et al., 2005). Early theoretical accounts suggested that serotonin facilitates behavioural inhibition (Deakin & Graeff, 1991; Soubrié 1986). More contemporary theories implicate serotonin in inhibiting behaviour in response to aversive predictions (Boureau & Dayan, 2011; Crockett, Clark, Apergis-Schoute, Morein-Zamir, & Robbins, 2012; Crockett, Clark, & Robbins, 2009; Dayan & Huys, 2009) and in regulating patience and impulsivity, thus facilitating long-term optimal behaviours and suppression of impulsive behaviours (Miyazaki, Miyazaki, & Doya, 2012; Miyazaki et al., 2014), though these two accounts have not been fully integrated (Cools, Nakamura, & Daw, 2011). Recent work in animals (see Miyazaki et al., 2014) show that the serotonin system enhances the ability to wait in order to obtain future reward and avoid future punishment. Specifically, serotonergic neurons increase their tonic firing rate when rats await rewards such as food. This evidence implies that serotonin enhances a focus on longer term goals, although if the same process operates in humans, this could be any long-term goal.

In this study, we tested the hypothesis that serotonin promotes a focus on long-term goals in dietary choice. We manipulated serotonin in healthy volunteers using the highly selective serotonin reuptake inhibitor (SSRI) citalopram, which enhances serotonin neurotransmission by blocking its reuptake and prolonging its actions in the synapse. To probe the neurochemical specificity of serotonin in regulating dietary choice, we contrasted the effects of citalopram with those of atomoxetine, a selective noradrenaline reuptake inhibitor that boosts noradrenaline neurotransmission and has a similar side-effect profile as citalopram. There is evidence noradrenaline has a distinct effect on cognitive flexibility and control (Chamberlain et al., 2006; Hyman, 2011). This evidence suggests that noradrenaline promotes inhibitory control in a global fashion—across different cognitive domains and executive tasks, which involves sending signals to brain regions in order to stimulate task-relevant processing and suppress irrelevant processes (Aron, Dowson, Sahakian, & Robbins, 2003; Miller & Cohen, 2001; Overtoom et al., 2003). Therefore, noradrenaline has been implicated in control of executive function, but not value-based decision making.

In summary, of the distinct varieties of action control, noradrenaline has been implicated in executive control function (including suppression of unwanted responses), while serotonin has been implicated in the domain of value-based decision making (including modulation of goal values). Here, we tested the implications of these distinct cognitive mechanisms of action control for the regulation of dietary choice. This experiment investigated the differential contributions of serotonin and noradrenaline in dietary choice. Specifically, we examined choices between healthy and unhealthy foods, in a free-choice task (i.e. without an explicit goal to eat healthier food), to investigate how performance is affected by acute manipulations of the serotonergic and noradrenergic systems. If serotonin modulates the weight of long-term goals in value-based decision making, then increased serotonin transmission will enhance the focus on health considerations, which will result in an increased proportion of healthy food choices. In contrast, if noradrenaline is implicated in control of executive function, but not value-based decision making, then acute modulation of noradrenaline will not affect healthy food choice.

## Method

### Participants

The protocol was approved by the Cambridgeshire Research Ethics Committee. Thirty healthy subjects (13 males; mean age, 25.6 years) were screened for neurological and psychiatric disorders by a consultant psychiatrist; each subject gave written informed consent before participating in the study. Exclusion criteria were any history of cardiac, hepatic, renal, pulmonary, neurological, psychiatric or gastrointestinal disorders (including eating disorders), medication/drug use, and personal or family history of major depression or bipolar affective disorder. Participants were financially compensated £10 per hour. Two participants dropped out of the study before completing all three sessions, and a third participant was excluded from all analyses because of the experience of peripheral side effects (dizziness and nausea) that prevented the participant from completing the cognitive tasks in at least one session. The final analysis was carried out in 27 participants (11 males).

### General procedure

Participants attended three sessions at Addenbrooke’s Hospital in Cambridge, UK (at least 1 week apart) and received single doses of atomoxetine (60 mg), citalopram (30 mg), and placebo in a double-blind counterbalanced design. We selected doses that were clinically relevant according to established treatment guidelines for attention-deficit/hyperactivity disorder (atomoxetine) and obsessive-compulsive disorder and depression (citalopram; British National Formulary; www.bnf.org), and in line with a previous study in our laboratory (Chamberlain et al., 2006). At the start of each session, participants took the drug orally. The food-choice task was administered as part of a broader testing battery commencing 1.5 hours after capsule administration, and ending approximately 3.5 hours after capsule administration. The timing of cognitive testing was based on approximate peak plasma levels for atomoxetine and citalopram (1–3 hours post capsule administration; Jiang et al., 2013; Henning & Netter, 2002; Kragh-Sørenson, Overø, Petersen, Jensen, & Parnas, 1981; Matsui et al., 2012; Sauer et al., 2005) and prior neurocognitive studies (e.g. Chamberlain et al., 2006). We attempted to standardize participants’ baseline hunger levels by instructing them to eat a regular meal before attending the session and giving all participants a standard snack (apple) during the rest period. Participants also completed trait questionnaires during the rest period, including the Restrained Eating Scale (Herman & Polivy, 1975), which measures preoccupation with weight and eating behaviour. At the end of the third session, the participants were debriefed about whether they had any suspicions about the order of drug administration; participants did not indicate any insight into the type of drug they received on each session.

### Food-choice task

The task had two stages (see also the Appendix). In the first stage, participants rated the perceived healthiness and tastiness of foods. In the second stage, participants made choices between pairs of foods (see Hare et al., 2009, for a related task). In each testing session, participants were first presented with 10 food items, in random order, and asked to rate each item on the dimensions of health and taste on a visual analogue scale ranging from zero to 1,000 (for better granularity of the measurement, although the scale can easily be converted to 1–10 range). Table 1 shows the food products used in the choice task in each session. Five of these items were preselected to be *virtues* (higher on health) and the other five were *vices* (lower on health). Participants’ ratings across all conditions confirmed these distinctions—the average health and taste ratings across all drug conditions and food types are presented in Table 2.

**Table 1:** Food products used in the choice task in each session

Session	Virtues	Vices
1	Red apple	Biscuits
	Green grapes	Truffles
	Strawberry	Chelsea bun
	Prunes	Mill shortbread
	Fruit & Nut	Donut
2	Green apple	Chocolate biscuits
	Red grapes	Dark chocolate
	Cherries	Danish
	Muesli	Pain au chocolat
	Mixed nuts	Vanilla cupcake
3	Pear	Chocolate chip cookie
	Blue berries	Dairy milk
	Apricot	Victoria sponge
	Raisins	Blueberry muffin
	Granola bar	Chocolate cupcake

**Table 2:** Average ratings across the drug (citalopram, atomoxetine, placebo), attribute (liking and healthiness) and food (healthy and unhealthy) factors

Food	Attribute	Drug		
		Placebo	Atomoxetine	Citalopram
Healthy	Liking	373.89 (60.56)	383.10 (46.51)	385.16 (53.84)
	Healthiness	451.13 (39.16)	439.37 (43.81)	439.42 (46.04)
Unhealthy	Liking	385.93 (73.92)	370.03 (72.98)	377.21 (79.44)
	Healthiness	198.07 (43.93)	203.35 (53.54)	192.03 (53.11)

*Note.* Standard deviations in parentheses

Following the ratings, participants were presented with pairwise combinations of the 10 food items and asked to select between the two items. Sometimes two virtues were paired, sometimes two vices were paired, and sometimes a vice was paired with a virtue. Following each choice, participants were asked to rate how satisfied they were with their choice. Prior to the task, participants were informed that upon completion, one trial would be randomly selected and the participant would receive the item that they chose on that trial.

To avoid order effects, we used different versions of the task on each session (see Table 1), each with unique food items, so that on each session participants made choices with respect to different food items. Task version was counterbalanced across participants. Although this exact task has not been previously published, we note that it is very similar to another food-choice task that has been widely used (Hare et al., 2009). The participants’ behaviour (percentage choice of healthy food) was correlated across sessions—Sessions 1 & 2: *r*(27) = .42, *p* = .029; Sessions 2 & 3: *r*(27) = .61, *p* = .001; Sessions 1 & 3: *r*(27) = .54, *p* = .004—and there was a high interclass correlation coefficient, *r* = .75, *F*(26, 52) = 4.22, *p* < .001, which suggests good test–retest reliability.

## Results

We focused our analysis on trials where participants made choices between virtues and vices. Table 3 presents the proportion of healthy choices (i.e., choices for the virtue over the vice) across the three conditions.

**Table 3:** Proportion of healthy choices across drug conditions

Drug	Proportion of healthy choices	
	Mean	*SD*
Citalopram	0.60	0.30
Atomoxetine	0.54	0.31
Placebo	0.45	0.31

A generalised linear model (generalised estimating equations) was run with the proportion of healthy choices as the dependent variable, and Drug condition and Session as within-subjects factors. The test of model effects revealed that there was a significant main effect of Drug condition, Wald chi-square (2) = 7.95, *p* = .0188; and the parameter estimates revealed that, relative to placebo, citalopram was a significant positive predictor of healthy choice, *B* = .276, Wald (1) = 7.96, *p* = .0048. There was neither a significant main effect of Session, Wald (2) = 1.59, *p* = .4515, nor an interaction between Drug and Session, Wald (4) = 5.09, *p* = .2782; which shows that our design successfully avoided any order effects. In a pairwise, Bonferroni-corrected comparison between the drug conditions, the estimated marginal means for the effect of Drug revealed that on citalopram participants made a significantly higher proportion of healthy choices relative to placebo (*p* = .0048), while the proportion of healthy choices did not significantly differ between atomoxetine and placebo (*p* = .1531), or between atomoxetine and citalopram (*p* = .2575).

We also measured individual differences in eating behaviour using the Restrained Eating Scale (Herman & Polivy, 1975). This scale measures preoccupation with weight and eating behaviour, and participants with scores higher than 17 are classified as restrained eaters. In our sample, the mean score (+*SD*) was 9.1 (+3.6). One participant had a score of 18; and the results did not change when excluding this participant from the analysis—for example, similarly significant: main effect of drug condition, Wald chi-square (2) = 6.52, *p* = .0384; parameter estimate of the citalopram effect, *B* = .148, *Wald* (1) = 6.30, *p* = 0.0121; and estimated marginal means effect of citalopram relative to placebo, *p* = .0315.

We next investigated how the drugs affected health and taste ratings of virtues and vices using a repeated-measures general linear model, with rating as the dependent variable and Drug (citalopram, atomoxetine, placebo), Attribute (taste and health), and Food (virtue vs. vice) as within-subjects factors. The results revealed neither a significant main effect of Drug, *F*(2, 52) = 0.21, *p* = .810, nor an interaction between Drug and Attribute, *F*(2, 52) = 0.62, *p* = .544, or between Drug and Food, *F*(2, 52) = 0.28, *p* = .761, which suggests that the drugs did not impact participants’ ratings of the healthiness or tastiness of the food items. Crucially, there was a significant Food × Attribute interaction, *F*(1, 26) = 237.68, *p* < .001, where health ratings were higher for virtues than vices (*M*_virtue_ = 443.31, *SD* = 42.92; *M*_vice_ = 197.81, *SD* = 49.97), while taste ratings were not different for vices and virtues (*M*_virtue_ = 380.71, *SD* = 53.49; *M*_vice_ = 377.72, *SD* = 74.84). There was also a significant effect of type of Food (virtues, vices), whereby virtue foods were rated higher than vice food overall across both health and taste ratings, *F*(1, 26) = 146.09, *p* < .001; but this effect (across all drug conditions) was driven by the difference in the health attribute rather than the taste attribute. There was also a main effect of Attribute, *F*(1, 26) = 63.37, *p* < .001, where taste ratings were higher than health ratings overall. In summary, those results confirm that our selected stimuli were appropriately distinguished on the basis of their healthiness.

We assessed the impact of health and taste dimensions on choice by computing for each attribute the difference in ratings between the chosen and unchosen options (see Table 4). The repeated-measures analysis used Drug and Attribute (health vs. taste) as a within-subject factors, with the difference in ratings between the chosen and unchosen options as the dependent variable. There was a significant main effect of Attribute, *F*(1, 26) = 6.193, *p* < .05, where the chosen–unchosen difference for taste ratings (*M* = 68.29, *SE* = 6.83) was greater than the chosen–unchosen difference for health ratings (*M* = 24.15, *SE* = 15.42), suggesting that, overall, our participants weighted taste more strongly than health in their choices. However, there was also a significant interaction between Drug and Attribute, *F*(2, 52) = 6.577, *p* < .01. As Table 4 shows, there was little variation across the three conditions in terms of the taste (chosen–unchosen) rating differences, while the health (chosen–unchosen) ratings showed large disparities between the conditions. Citalopram showed the largest difference between ratings in the health dimension. This pattern implies that on citalopram, participants more frequently selected the option that had the higher health rating (i.e., more often the chosen food had a higher health rating than the rejected food).

**Table 4:** Difference between chosen and unchosen foods across healthiness and liking attributes for each drug condition

Drug	Liking difference (Chosen–Unchosen)		Healthiness difference (Chosen–Unchosen)	
	Mean	*SD*	Mean	*SD*
Citalopram	66.09	7.11	45.11	17.91
Atomoxetine	67.81	8.71	34.63	17.50
Placebo	70.98	7.97	-7.30	19.54

One possible alternative explanation for our findings is that citalopram did not affect decisions by modulating goal values per se, but rather made participants less hungry, which in turn made them less likely to select the vice. To test this, we first examined whether citalopram and atomoxetine affected hunger ratings. Baseline hunger levels were moderate across all conditions (*M*_citalopram_ = 4.59, *SD* = 1.42; *M*_atomoxetine_ = 4.82, *SD* = 1.21; *M*_placebo_ = 5.46, *SD* = 0.97). A repeated-measures general linear model with hunger as the dependent variable and Drug (citalopram, atomoxetine, placebo) as a within-subject factor revealed a significant main effect of Drug, *F*(2, 25) = 6.56, *p* = .005. Tests of within-subjects linear contrasts revealed that both citalopram, *F*(1, 26) = 12.43, *p* = .002, and atomoxetine, *F*(1, 26) = 6.57, *p* = .017, reduced the ratings of hunger relative to placebo. The same pattern was exposed by Bonferroni-corrected pairwise comparisons between the marginal means for citalopram and placebo (*p* = .005), atomoxetine and placebo (*p* = .050), but not citalopram and atomoxetine (*p* = 1.000). Decreased appetite is a clinically well-known side effect of atomoxetine (Michelson et al., 2003), but not of citalopram (Khawam, Laurencic, & Malone, 2006). Furthermore, given that only citalopram affects choice, we tested whether individual differences in the citalopram effect on hunger correlated with the citalopram effect on choice (i.e. those subjects who showed stronger citalopram effects on hunger also showed the stronger citalopram effects on choice). This correlation was indeed negative and significant, *r*(27) = -.42, *p* = .029, which warranted a mediation analysis (Judd, Kenny, & McClelland, 2001) to test whether changes in hunger caused by citalopram mediated drug-induced changes in self-control. In the mediation analysis, the direct effect of citalopram on choice was again significant, *t*(26) = 2.51, *p* = .0186. The indirect (mediation) effect of citalopram on choice through hunger was only marginally significant (*Z* = 1.75, *p* = .081). Therefore, citalopram’s effects on food choice could partially be attributed to changes in appetite.

Finally, citalopram-induced changes in dietary choice could be caused by nonspecific side effects, such as nausea, of citalopram (Khawam et al., 2006). In our study, we also asked the participants to rate their experience of eight ‘mood’ states in every condition. We ran a multivariate ANOVA (GLM) with Mood ratings as the dependent variable and Drug as the within-subjects measure. All mood ratings were insignificant, apart from “nauseous”: positive affect: *F*(2,81) = 1.572, *p* > .05; negative affect: *F*(2, 81) = 1.290, *p* > .05; drowsy: *F*(2, 81) = .570, *p* > .05; hostile: *F*(2, 81) = .390, *p* > .05; irritable: *F*(2, 81) = .195, *p* > .05; energetic: *F*(2, 81) = .593, *p* > .05; attentive: *F*(2, 81) = .967, *p* > .05; nauseous: *F*(2, 81) = 6.079, *p* = .003. Planned comparisons showed that nausea was higher in the atomoxetine (*M* = 65.61, *SD* = 98.34) than in the citalopram (*M* = 31.46, *SD* = 86.74) and the placebo (*M* = -11.11, *SD* = 87.39) groups, respectively. We ran a mediation analysis of the citalopram versus placebo effect on choice, with nausea as the mediator, but the mediation effect was not significant (*Z* = 0.99, *p* = .324), while the total effect of citalopram on choice was significant, *t*(26) = 2.51, *p* = .0186.

## Discussion

We found that acute citalopram increased choices for healthy foods relative to placebo, and this appeared to be driven by a greater emphasis on the health, rather than taste, attributes of food items. In contrast, atomoxetine did not appear to significantly affect the proportion of healthy choices relative to placebo. Citalopram did not significantly impact food choice relative to atomoxetine, however. Thus, our data tentatively suggest that serotonin amplifies the importance of health considerations in consumption decisions, perhaps via enhancing a focus on, or the value of, long-term outcomes. The lack of a direct effect of the drug on health or taste ratings of food items also suggests serotonin promotes integration of health information into choices rather than influencing the health ratings themselves. This result also supports the standard hypothesis that serotonin can facilitate behavioural inhibition (Boureau & Dayan, 2011; Deakin & Graeff, 1991; Soubrié, 1986), as a result of impacting the weighting of health outcomes or goals in value-based choice.

The effect of citalopram was only partially mediated by a reduction of appetite (i.e. the participants were more likely to select virtue foods, and reject vice foods, when they were less hungry). This marginal mediation implies that serotonin may impact cognitive processes (i.e. a focus on health), appetite, as well as the interaction between appetite and cognition (i.e. a reduced appetite may focus one’s attention on the value of health and long-term goals in general). The finding that atomoxetine also reduces hunger, but does not significantly affect choice, supports the interpretation that citalopram may have an independent effect on cognitive processes.

There is an extensive literature on food choice following serotonin releasing drugs such as fenfluramine (Wurtman & Wurtman, 1979; Wurtman et al., 1985). Specifically, Wurtman’s early data and theories reveal how serotonin shifts food choice from carbohydrate to protein—serotoninergic drugs depress food intake and carbohydrate consumption without impacting protein consumption. Other researchers have tended to focus on more dietary aspects of the effects of serotonergic regulation (see Blundell, 1992). This research reveals that serotonin in the body mediates nutritional input and the feeding drive. Specifically, manipulation of serotonin causes changes in feeding behaviour, such as when serotonergic activation leads to selective avoidance of fat in the diet (Blundell, Lawton, & Halford, 1995); whereas serotonergic activation is modulated by nutritional variables such as the proportion of carbohydrate, the availability of specific macronutrient sources, the degree of hydration, and the circadian preference for specific nutrients (Blundell, 1992). This literature on food choice could, in principle, explain some of the observed effects on choice in our study—specifically, the reduced preference for vices, because those foods predominantly contain carbohydrate and fat (see Table 1). However, this literature cannot account for the observation that the citalopram’s effect is driven by a greater emphasis on the health rather than taste attribute in choice, specifically the effect on the frequency of selecting the option that has higher health rating. Therefore, we conclude that serotonin can impact dietary decisions by enhancing the impact of health information and long-term goals.

In terms of a mechanistic interpretation of our findings, we speculate that serotonin modulates the functional connectivity between ventromedial prefrontal cortex (vmPFC) and dorsolateral prefrontal cortex (DLPFC). This interpretation is motivated by evidence that preference for healthier food items in similar dietary decision tasks is linked to increased functional connectivity between vmPFC and DLPFC regions encoding foods’ healthiness (Hare et al., 2009; Maier, Makwana, & Hare, 2015). Note that although those studies were not concerned with the role of serotonin, they nevertheless employed a very similar dietary decision task. Therefore, such connectivity changes could also provide a plausible mechanism for integrating health information into food choice under the increased influence of serotonin. This interpretation is consistent with Miyazaki et al.’s (2012) hypothesis that serotonin enhances prefrontal regulation of action, most likely through structures such as the medial prefrontal and orbitofrontal cortices, which are involved in value-based decision making. The current data demonstrate that this effect of serotonin occurs not only during the regulation of behaviour whilst waiting for (food) reward (Miyazaki et al., 2014) but also during other self-control problems such as (food) choice. In summary, our results may shed light on the underlying neurochemical substrates involved in self-control.

We acknowledge the small sample size as a key limitation of this study, given the observed effects are relatively weak, which invites the need for future replications with larger samples and more heterogeneous populations (healthy participants as well as individuals undergoing weight-loss treatment).

The findings also have implications for understanding and treating health conditions such as eating disorders and obesity. For example, serotonin enhancing drugs could be given during initial stages of behaviour change interventions aiming to change the lifestyle of overweight individuals and obese patients. Similarly, the affected cognitive processes also have important implications for disorders of human decision making such as addiction and impulsive behaviours. Pharmacological treatments that enhance the weight of long-term goals in value-based decision making might support psychological therapies for such mental health conditions. Finally, targeting the serotonin system and its impact on value computation in decision making offers the potential for better understanding consumer food preferences and behaviour.

## Appendix

### Methodological details

The task was run in E-Prime software (Schneider, Eschman, & Zuccolotto, 2012).

Participants saw the following instructions:

- “You will now see a series of items. Please assign a rating to each item by clicking the mouse once on the provided scale. If you can’t tell what an item is, please notify the experimenter.”
- “You will now see pairs of items. Please select which item you would like to eat at the end of the experiment. Click the left mouse button to select the item on the left, or click the right mouse button to select the item on the right.”
- “After you have made your decision, you will be asked to rate how satisfied you feel with your decision.”
- “One of these trials will be selected by the computer at the end of the task and you will receive the item you chose in that trial, so treat each choice as if it were the only one.”

Participants saw the following stimuli:
